# Variations in the Presentation of Aphasia in Patients with Closed Head Injuries

**DOI:** 10.1155/2010/678060

**Published:** 2010-03-02

**Authors:** Dara Oliver Kavanagh, Conor Lynam, Thorsten Düerk, Mary Casey, Paul W. Eustace

**Affiliations:** ^1^Department of Surgery, Mayo General Hospital, Castlebar, Co Mayo, Ireland; ^2^Department of Radiology, Mayo General Hospital, Castlebar, Co Mayo, Ireland

## Abstract

Impairments of speech and language are important consequences of head injury as they compromise interaction between the patient and others. A large spectrum of communication deficits can occur. There are few reports in the literature of aphasia following closed head injury despite the common presentation of closed head injury. Herein we report two cases of closed head injuries with differing forms of aphasia. We discuss their management and rehabilitation and present a detailed literature review on the topic. In a busy acute surgical unit one can dismiss aphasia following head injury as behaviour related to intoxication. Early recognition with prolonged and intensive speech and language rehabilitation therapy yields a favourable outcome as highlighted in our experience. These may serve as a reference for clinicians faced with this unusual outcome.

## 1. Introduction

Closed head injury may result in a wide range of speech and language deficits ranging from total loss of interpersonal communication to minor flaws in word selection. They constitute a significant workload in the acute hospital setting. 

Aphasia is a recognised yet under reported sequela of closed head injury. It is defined as impairment of comprehension or production of language in written or spoken forms due to an acquired lesion of the dominant cerebral hemisphere. There are many types of aphasia ranging from nonfluent (Broca's) aphasia, which is characterised by slow and incorrectly articulated speech to fluent (Wernicke's) aphasia in which the patient exhibits well-articulated speech, which lacks meaning. Broca's area as described by the French pathologist Pierre Broca lies in areas 44 and 45 of Brodman in the inferior frontal gyrus of the dominant cerebral hemisphere, usually the left cerebrum. Injury to this area will produce a Broca's aphasia [1]. Broca's (motor) aphasia is associated with a deficit in speech production or language output, often accompanied by a deficit in written communication. The patient is aware of the impairment. Wernicke's area, as described by the German Neurologist Karl Wernicke, lies in the posterior part of area 22 in the superior temporal, gyrus of the left cerebral hemisphere. Wernicke's (sensory) aphasia is characterised by impaired comprehension of spoken and written words, associated with effortless, articulated but paraphasic, speech and writing. Circumlocution can be a feature. More aggressive forms are characterised by incomprehensible speech. The patient is frequently unaware of the deficit. The speech centres are linked by the arcuate fasciculus. This curves around the posterior end of the lateral fissure within the white matter deep to the supramarginal gyrus [2]. 

The current series represents the experience of a district general hospital. Over 2000 annual referrals are evaluated in the emergency department. Of these, two hundred and seventy-one (range 251–317) met the criteria for acute admission to the Surgical Unit based on Advanced Trauma Life Support (ATLS) guidelines. Nine (range 9–12) patients were transferred to the National Neurosurgical Centre at Beaumont Hospital for more detailed neurosurgical management. Herein, we report two differing forms of aphasia following significant closed head injuries with favourable outcomes.

## 2. Case  1

A 27-year-old english-speaking right-handed Irish male was involved in a road traffic accident in which he was projected thirty meters from his motorbike following a head-on collision with an approaching vehicle at 1700 hours. He was wearing a helmet and full protective clothing. On arrival in the accident and emergency department at 1830 hours his Glasgow coma scale (GCS) was 4/15. He was intubated and ventilated. His pupils were equal and reactive to light. His vital signs were within normal limits. He had bilateral periorbital haematomas (Racoon eyes) and a right-sided hemiparesis. 

**Figure 1:**
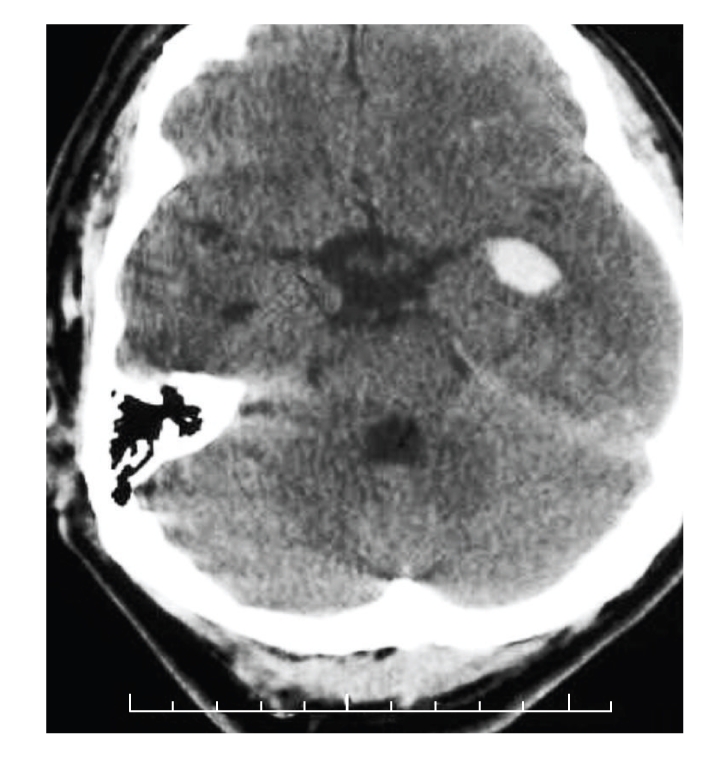
There is an area of high attenuation involving the left temporal lobe anterosuperiorly corresponding with Wernicke's area.

Computed Axial Tomography (CT) scan as shown in Figure 1 revealed an intracerebral haemorrhage in the left temporal lobe corresponding to Wernicke's area. Other radiological findings included fractures of the base of the right thumb, the right radial styloid, the right ulnar styloid, the radial and ulnar diaphyses, and comminuted fractures of the right humerus and femur. Following discussion with the National Neurosurgical Centre he underwent operative fixation of his orthopaedic injuries. He was managed initially in the intensive care unit and was gradually weaned off sedation. His GCS returned to 15/15 on the 16th day. He had significant expressive difficulties (Wernicke's aphasia). His speech was fluent but contained paraphasias and jargon. He was unaware of these abnormalities. He was unable to read text aloud and understand written language. A speech and language assessment reported severe comprehensive difficulties. The patient gave inaccurate yes/no answers to either personal or general orientation questions. He required extra-verbal cues to facilitate auditory comprehension. His lack of awareness of his reduced communicative abilities resulted in frustration. It was difficult to ascertain whether semantic memory compromise was evident. At the time of discharge his receptive aphasia had improved slightly and he was referred for further speech and language rehabilitation. A formal psychological evaluation was unavailable. He was seen at two-week intervals by the speech therapist. His speech and language improved but he demonstrated decreased attention and tangential speech with compromised reading ability (40% reading comprehension of single words). He also had a significant reduction in fluency with pronunciation difficulties and circumlocution. His receptive function was unaffected. His short-term memory was also impaired. Categorisation and odd-one-out activities were used to stimulate semantic processing and representations. At six-month follow-up his fluency of speech had improved significantly. He was able to construct complete sentences with occasional circumlocution. His comprehension of single words improved to 90%. He produced errors only during higher level semantic tasks. He continues to improve.

## 3. Case  2

**Figure 2:**
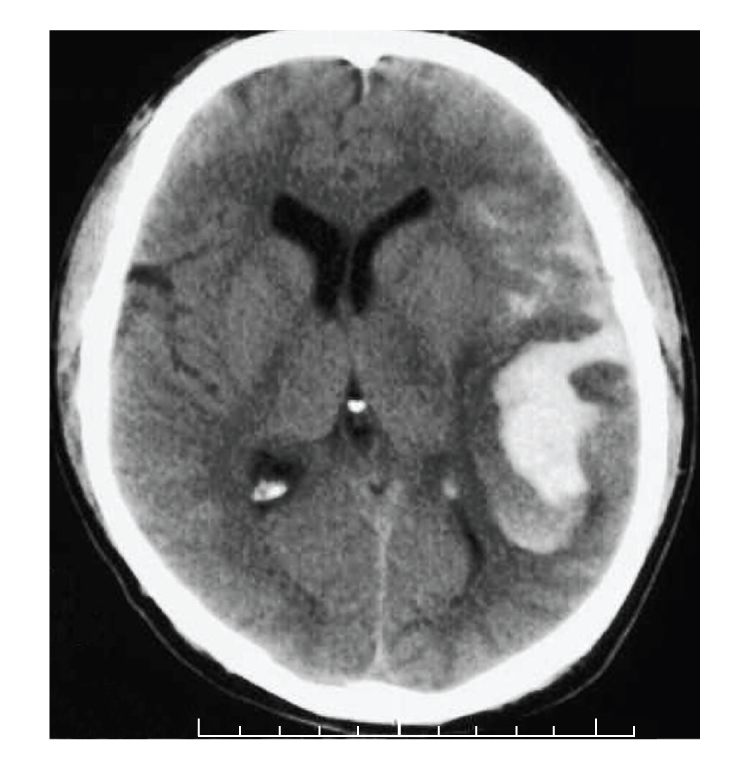
There is a large area of high attenuation involving the left frontal, temporal and parietal lobes incorporating Broca's area with subarachnoid extension and fresh haemorrhage in the suprasellar cistern.

A 32-year-old english-speaking right-handed intoxicated Irish male presented following a fall onto a concrete path with a GCS score of 9/15. Both pupils were equal and reactive to light. His blood alcohol levels were 383 mcg per litre, which clouded the clinical picture (a serum alcohol level >80 mcg per litre is equivalent to 2 units of alcohol). His vital signs were within normal limits. He had multiple facial abrasions and a soft tissue swelling on the left occipital region and blood was seen emanating from his left ear. Auroscopy revealed haemotympanum. He was commenced on prophylactic intravenous antibiotics. CT brain (Figure 2) demonstrated a haematoma of the left frontal, temporal and parietal lobes superimposed upon a previous haematoma along with subarachnoid extension with slight midline shift to the right and evidence of air in the posterior cranial fossa. The National Neurosurgical Centre recommended supportive therapy and serial clinical assessments. The following morning his GCS deteriorated to 7/15 and he was intubated and ventilated. A right-sided hemiparesis was noted. His pupils and haemodynamic parameters were unchanged. A follow-up CT brain was unchanged. He was transferred to National Neurosurgical Unit in view of this clinical deterioration. A craniotomy was performed with evacuation of the temporal lobe haematoma and insertion of an intracranial pressure monitor. He was extubated on the 14th postoperative day and initially obeyed commands. He returned to our institution for further rehabilitation. Initially he had features of a motor aphasia (Broca's). He was able to respond nonverbally by blinking his eyelids. Assessment of auditory comprehension was difficult but appeared intact. As his clinical condition improved his expressive capabilities improved but did not fully recover. After 12 weeks he successfully made his first verbal response. He was transferred to the National Rehabilitation Centre. At 3-month follow-up he had ongoing circumlocution and reported difficulty with reading and writing. He reported persistence of posttraumatic amnesia. His mobility problems related to hemiparesis had resolved and he was independent in all activities of daily living.

## 4. Discussion

Aphasia is the primary language abnormality in adults and has multiple causes including trauma, infection, brain tumours, hypoxia, and obstructive hydrocephalus. Although the association between language disorders and head trauma has been known for millenniums, descriptions of such cases were scarce until World Wars I and II, when head injuries were, for the most part, penetrating wounds [1]. In contrast with these aphasias secondary to penetrating wounds, there are still few detailed descriptions of aphasia after closed head injuries. They can be challenging clinically. The absence of significant other neurological signs can result in cases being misdiagnosed as a transient ischemic attack or cerebrovascular accident [3]. They can be dismissed as intoxication, confusion, and psychosis or even malingering. In particular it has been noted that fluent aphasia can be elusive to nonneurologically orientated physicians [4]. The availability of a neurologist allows detection of more subtle deficits. The incidence of language disturbance is 1 per 1000 emergency referrals. They highlight the clinical challenge one is sometimes faced with when initially evaluating the intoxicated head injured patient. They reinforce the need to determine the mechanism of injury and thereby an appropriately high index of suspicion. The cases presented in this paper illustrate clear examples of both motor and sensory aphasias associated with a closed head injury and provide a guide to clinicians as to the presentation, management, and prognosis of such cases. 

The severity of head injury can be scored on numerous parameters such as GCS on arrival, neurological deficit, and/or CT findings. One of the most consistent prognostic markers used in the medical literature is the presence of posttraumatic amnesia (PTA), where PTA for longer than one week predicts a severe head injury. There has been a growing debate in the neuropsychiatric literature on the question of whether patients who sustain a blunt head injury can develop symptoms of acute stress disorder (ASD) and posttraumatic stress disorder (PTSD). These are typically characterised by fear and intrusive recollections of the event. Speech deficits do not tend to be a major component of these sequelae. Over the last few years there have been many publications in the field of military medicine reporting cases of mild traumatic head injury due to blasts experiencing [5–7]. PTSD is less evident in the setting of severe head injury as described in the current series. Data disputing an association between traumatic brain injury and PTSD comes from Warden et al. among others [8]. He examined 47 patients with moderately severe TBI and found that although a minority of subjects endorsed the avoidance and autonomic arousal symptoms of PTSD, none reported any reexperiencing. A study of victims of road traffic accidents reached similar conclusions after finding a dearth of PTSD symptoms in a subgroup of patients who had briefly lost consciousness [9]. Feinstein et al. report symptoms of PTSD in a sample of head-injured patients assessed within a couple of months of injury and stratified for severity of head injury on the basis of duration of PTA. However, when PTA extended beyond one hour, symptoms of reexperiencing the traumatic event (intrusive phenomena) and avoidant behaviour occurred significantly less often. We need to recognize that irrespective of the mechanism involved, symptoms of PTSD may occur across the full range of head injury severity and demand our prompt attention [10]. 

Subtle changes in speech can also be evident. The spontaneous speech of head injured patients can be inappropriate in length, confused, lacking informational content, and slow. Language deficits may not be the sole cause for impairments in discourse. Normal discourse also requires adequate social and cognitive abilities, and these abilities are frequently impaired in closed head injured patients. The prognosis for recovery of language functioning is good but residual deficits, especially in naming, may persist over many years. Subjective reports of language problems in head injured patients are not as frequent as the reports of memory problems. 

Aphasia associated with head injury can develop hours or even weeks after the initial precipitating event. It is most commonly associated with right blunt orbito-frontal trauma with a contre-coup left temporo-parietal injury [3, 11, 12]. This type of impact imparts a shearing force to the head which causes motion of the cerebral hemispheres within the closed cranium. Peripheral areas are more susceptible to direct impact with the cranium. 

Menon et al. describe their experience with 31 consecutive patients with closed head injuries over a two-year period. The mean age was 36 years old. They demonstrated that poor outcome in relation to speech occurred more frequently in the presence of severe head injury (GCS < 8) on arrival or a prolonged comatosed state (>132 hours). Both patients in the current series had similar ages and had GCS scores of 4 and 9, respectively. Paradoxically case 1 (GCS = 4) had a better functional outcome. However, case 1 was comatosed for 72 hours while case 2 was comatosed for 168 hours perhaps explaining the differing functional outcomes [13]. Overall, communication disability tends to recover well especially during the first 6 months and to a lesser extent over the next three years regardless of severity of the injury [14–16]. Dahlberg et al. identified a volunteer sample of 52 people with closed head injury who were at least 1 year post injury [17]. They had received rehabilitation and had persistent social communication deficits one year post injury. Patients were randomised to receive treatment or not. Subjects who received social communication skills training had improved communication skills that were maintained on follow-up. Overall life satisfaction for participants was improved. In fact, it has been suggested that recovery can continue up to 10 years [18]. Few cases recover their pre-morbid status. Alajouanine et al. report a more favourable outcome with posttraumatic aphasias than with aphasias related to cerebrovascular disease. 

Even mild brain injury (GCS > 12) can result in subtle long-term impairment of speech. Whelan et al. compared a detailed language profile of a 19-year-old woman, 2 years following a mild brain injury with a matched normative cohort of 10 participants with no neurological impairment. Deficits in attention, lexical access, complex lexical-semantic manipulation, response monitoring, and organization were revealed. Thus lending support for the hypotheses pertaining to neuronal fallout mechanisms within the frontal lobes as a consequence of mild neurological insult [19]. Others have proposed that long-term speech deficits and other functional deficits have therapeutic potential other than speech therapy. It has been hypothesised that “idle” or “recoverable” injured neurons may be evaluated with SPECT imaging prior to administration of hyperbaric oxygen. These were subsequently evaluated with SPECT imaging post therapy, demonstrating perfusion. This hypothesis has not gained popularity despite initial enthusiasm. Early goal directed therapy in the treatment of the trauma patients with adequate cerebral oxygenation and prevention of hypotension is paramount to ensure a patient reaches a stage, where rehabilitation is possible [20]. Future work is essential to optimise outcome from this socially debilitating deficit as the incidence of blunt trauma due to motor vehicle injuries continues to rise.

## 5. Conclusion

Patients who sustain closed head injuries are susceptible to a variety of functional and psychological impairments. Speech is one of the cardinal factors that determine the outcome of a prolonged illness and intensive rehabilitation. It determines whether one can integrate into daily life at home and at work. These cases highlight that despite significant initial deficits in speech and language following closed head injuries favourable long-term outcomes can be achieved with prolonged and intensive rehabilitation.
